# Psychometric evaluation of a national exam for clinical undergraduates

**DOI:** 10.3389/fmed.2022.1037897

**Published:** 2022-12-14

**Authors:** Yuting Han, Zhehan Jiang, Jinying Ouyang, Lingling Xu, Ting Cai

**Affiliations:** ^1^Institute of Medical Education, Peking University, Beijing, China; ^2^National Center for Health Professions Education Development, Peking University, Beijing, China; ^3^Graduate School of Education, Peking University, Beijing, China

**Keywords:** medical examination, psychometrics, item response theory (IRT), quality control, item analyses, test information

## Abstract

**Background:**

As a high-stake national-level examination administrated before students’ clerkship in China, the Standardized Competence Test for Clinical Medicine Undergraduates (SCTCMU) has received much attention from the relevant educational departments and society at large. Investigating SCTCMU’s validity and reliability is critical to the national healthcare profession education.

**Materials and methods:**

Raw responses from SCTCMU, answered by 44,332 examines of 4th-year undergraduate medical students on 300 multiple-choice items, were used to evaluate the quality of the exam *via* psychometric methods based on item response theory (IRT). The core assumptions and model-data fit of IRT models were evaluated, as well as the item properties and information functions.

**Results:**

The IRT models were fitted to the observed assessment data, where all the required assumptions were met. The IRT analysis showed that most items had acceptable psychometric properties, and the passing score was located close to the lowest measurement error computed from the model outcomes.

**Conclusion:**

The proposed modern psychometric method provides a practical and informative approach to calibrating and analyzing medical education assessments. This work showcases a realistic depiction of the IRT analysis process and therefore facilitates the work of applied researchers wanting to conduct, interpret, and report IRT analyses on medical assessments.

## 1 Introduction

The Standardized Competence Test for Clinical Medicine Undergraduates (SCTCMU) is a summative assessment administrated prior to students’ clerkship in China. It consists of 300 dichotomously scored multiple-choice items measuring knowledge of 13 pre-grouped systems (e.g., immune system and endocrine system) crossed three cognitive levels-*Memory*, *Understanding*, and *Application*. After 10 years of pilot investigation at the regional level, SCTCMU, likely the largest-scale examination of its kind worldwide (i.e., national standardized tests for clinical undergraduates), was formally launched in Spring 2021, where 99 medical schools participated, tallying up above 44,000 undergraduates majoring in clinical medicine. As a large-scale national examination, SCTCMU’s product development life cycle and its operation and management are strictly regulated to abide by the standards of the national licensing exam. It’s, however, unknown that if using these standards are appropriate for the target population that are not qualified yet for the profession. Positive results are surely inspiring as assessment consistency across education phrases (i.e., undergraduates to licensures) can be testified. On the other hand, inappropriate setting are likely to result in poor assessment and incorrect diagnostic feedback for both items and examinees.

The quality of SCTCMU is undoubtedly of great importance and needs to be validated. Most studies on medical education tests are analyzed and explained based on classical test theory (CTT), which is easy to understand, simple to operate and integrated into a complex system of analysis (1–4). However, quality indicators of CTT models (e.g., item difficulty and discrimination) are sample-dependent (5–7), resulting in repercussions due to the change of test takers. With the development of psychological and educational measurement, item response theory (IRT) has been used for large-scale assessment evaluation and quality control. IRT is a critical technique that estimates examinee proficiency and item difficulty on the same scale. When an IRT model fits with observed assessment data and all the statistical assumptions are met, the vexing problem of sample dependency is eliminated, and the precision of each ability estimate becomes computationally available (8). IRT models are widely used worldwide for evaluating assessments in medical education, including some medical specialty board certifying examinations and professional licensure examinations. For example, the National Board of Medical Examiners (NBME) uses the Rasch model- a special case of IRT models- to calibrate its tests and provide fundamental psychometric information to item writers and content developers (9).

This paper aims to use psychometric methods to evaluate the quality of SCTCMU whose target population is 4th-year medical undergraduates. Studies of this kind are valuable in the following aspects: (a) rare large-scale examinations are designed specifically for undergraduate medical education and, therefore, a successful case may serve an appropriate reference for other large-scale assessment providers globally, (b) the proposed workflow provides a deeper analysis giving multifold information on the behavior of individual items and examinees as well as the underlying constructs being examined, and the step-by-step demonstration makes this paper an illustrative guide for similar studies, as an alternative to traditional CTT analyses; (c) using advanced modeling strategies to support developing valid and reliable tests of student performance is critical to both improve assessment quality and maintain curricula standards of fairness and objectivity; (d) the testing standards used in higher-level assessment can be tailored to population with lower-level competence if skill/knowledge consistency indeed exists.

Correspondingly, the paper includes three components: (a) examining whether SCTCMU scores discriminate against students of different abilities, (b) justifying the validity of the cutoff score set by subject matter experts, and (c) provides details on the IRT analysis process for medical education researchers.

## 2 Materials and methods

### 2.1 Data collection

Standardized Competence Test for Clinical Medicine Undergraduates is jointly developed and administrated by the China National Medical Examination Center and the National Center for Health Professions Education Development; the test is designed to assess students’ level of competence at the end of the 4th-year of their 5-year undergraduate studies in clinical medicine. It is based on the content specified in the National Chinese Medical Qualification Examination Standards to determine the scope of content; Corresponding proportions in each subject are shown in Table 1. It consists of 300 dichotomously scored multiple-choice items with four distractors and a single key. Raw responses of 44,332 were used as the source data.

**TABLE 1:** Summary of content and scope of Standardized Competence Test for Clinical Medicine Undergraduates (SCTCMU).

Subject	Content	Key points	Proportion
Preclinical medicine	anthroponomy, biochemistry, physiology, pathophysiology, microbiology, immunology, pathology, pharmacology	understanding and application of knowledge related to human health and disease	40∼45%
Medical humanities	medicopsychology, medical ethics, sanitary legislation	basic knowledge and important principles	5∼10%
Preventive medicine	basic concepts of preventive medicine and tertiary prevention of diseases, statistical methods in clinical medicine, basic principles and methods of clinical epidemiology, the impacts of environmental factors on health	important concepts, basic principles and applications in disease prevention and control	5∼10%
Clinical medicine	diagnostics, internal medicine, surgery, obstetrics and gynecology, pediatrics	symptoms and signs, etiology and pathogenesis, diagnosis and differential diagnosis, principle of treatment	40∼45%

### 2.2 Overall workflow

**FIGURE 1:**
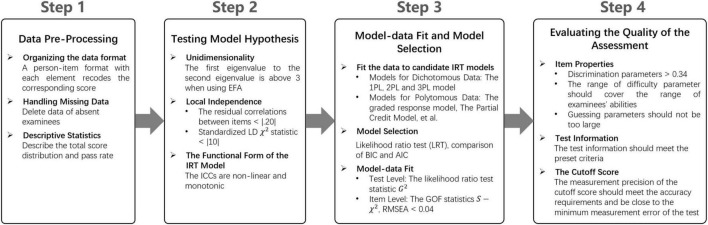
The steps of using psychometric methods to evaluate the quality of medical education assessments.

To conduct the psychometric evaluation of SCTCMU, a 4-step flow covering essential tasks was adopted, as seen in Figure 1. Without statistical and/or mathematical, one can regard a step as a pre-requisite of its descendant, and rules of thumb for all necessary statistics (i.e., thresholds) are also appended in the Figure. Step 1 is self-evident that ensuring the target dataset’s quality is a foundation for further analysis, and its descriptive statistics, although not advanced modeling, provide a general view of the assessment. In the second step, all assumptions should be satisfied so that IRT models can be constructed, or the assessment should be evaluated through other alternative methods. Like all statistical modeling procedures, Step 3 drives researchers to check the appropriateness of the model, including which candidate model performs the best and the quantitative values for model fit evaluation. After confirming the use of IRT, the final step is extracting information yielded from the selected model and interpreting the results, including item- and test-level properties and pass/fail decisions of the examination. Details about each point listed in the steps are described below.

### 2.3 Statistical analyses

The IRT approach was used to explore the psychometric properties of the test. The IRT models specify the respondent’s probability of a correct answer on a test item based on both a person’s parameter θ_*j*_ and a vector of item characteristics. The three-parameter logistic (3PL) IRT model was defined as follows (10):


(1) $$P\,\left(\theta_{j}\right)=c_{i}+\left(1-c_{i}\right)\,\frac{1}{1+e^{-D\,a_{i}\,\left(\theta_{j}-b_{i}\right)}}$$

where *b_i_* is the difficulty of the item *i*, which is on the same scale as the ability parameter, whereas *a_i_* is an item discrimination parameter (or item slope) determining how well the item discriminates between subjects with different abilities. “Good” discrimination parameters range from 0.8 to 2.5 (11). *c_i_* is often referred to as a pseudo-guessing parameter (or lower asymptote) representing the contribution of guessing to the probability of correct response, or someone with infinite low ability has a certain probability of scoring 1 on item *i*. And *D* is a constant usually taking a value of 1 or 1.7, indicating the use of a logistic scale, or an approximated normal scale, respectively. Other dichotomous logistic IRT models can be seen as special cases of the 3PL model. For instance, the two-parameter logistic IRT (2PL) model is obtained by setting all *c_i_* to be equal to 1. In contrast, the one-parameter logistic IRT (1PL) model or Rasch model additionally sets all *a_i_* to be equal to 1.

#### 2.3.1 Core assumptions

A set of assumptions must be met in IRT measurement, including unidimensionality, local independence, and the functional form (i.e., the item response functions) of the IRT model (12–15).

Factor-analytic methods are commonly used to test whether a one-dimensional construct is underlying the exam. In the statistical literature, it’s said that if the ratio of the first eigenvalue to the second eigenvalue is above 3 when using exploratory factor analysis (EFA), the single dimension assumption will be satisfied (16).

Local independence assumes that responses to different items are not related to each other when the latent trait is held constant. The residual correlations and standardized LD χ^2^ statistic (17) between items are inspected to detect local independence. Residual correlations greater than |0.20| are considered possible local dependence (15, 18). LD statistics between |5| and |10| indicate moderate and questionable LD; greater than |10| reflect likely LD issues (17).

The item response function of IRT model allows the construction of item characteristics curves (ICC), which describe the relationship between the probability of correctly answering an item and the target ability level (19). When dealing with dichotomous data, the ICC is generally assumed to be non-linear and monotonic. The assumption of monotonicity implies that the probability of answering an item correct should increase along with the raise of latent ability. Test characteristic curve (TCC) is interpreted similarly to the ICC, except that the *y*-axis is replaced with “expected score” (i.e., performance on the observed score scale but estimated by a model).

#### 2.3.2 Model-data fit and model selection

After the assumptions above were tested and satisfied, the Rasch model, 2PL model and 3PL model were fitted to the data and compared to each other, allowing to select the simplest and best-fitting model. The likelihood ratio test statistic *G*^2^ was used to examine the test level model-data fit. A likelihood ratio test (LRT), the relative change statistic [$R_{{△}_{2}}=\frac{-2\,L\,L\,\left(\mathrm{Reduced}\,\mathrm{model}\right)-\left[-2\,L\,L\,\left(\mathrm{Full}\,\mathrm{model}\right)\right]}{-2\,L\,L\,\left(\text{Re}\,\mathrm{duced}\,\mathrm{model}\right)}$; (20)], Bayesian information criterion (BIC), and Akaike information criterion (AIC) were used for model comparation. After selecting the best fitting model, the goodness-of-fit (GOF) statistics S-χ^2^ (21–23) was adopted to assesses how far the observed data match those expected by the IRT model at the item level. Items with a significant S-χ^2^ value indicates the model does not fit a given item (15, 24). When the sample size is large, the GOF indexes based on statistical test are prone to be very sensitive in terms of rejecting the fitted model, although the degree of misfit may be practically tolerable or trivial (25). Therefore, the root mean square error approximation [RMSEA; (26)] was used to gauge the magnitude of misfit, and the recommended upper limit is 0.04.

#### 2.3.3 Item properties

Essential IRT analyses are presented as item difficulty and item discrimination. The items can be evaluated based on some cutoff values. Items with discrimination values less than 0.8 should be excluded (27, 28). Baker (29) further divides the discriminative ability of an item into none (0), very low (0.01 to 0.34), low (0.35 to 0.64), moderate (0.65 to 1.34), high (1.35 to 1.69), very high (above 1.70), and perfect (+ infinite) according to its discrimination value. And the values of the *b* parameter should be in the range of (−3, 3) (30).

#### 2.3.4 Information functions

Item information functions (TIF) describe the contribution of test items to the assessment of examinee ability at various ability levels. The sum over all items gives the test information function (TIF), which indicates how much information is collected by the entire test across the ability range and makes it possible to estimate at which ability level the test is most accurate. Besides, the standard error of measurement in IRT is inversely proportional to the square root of the test information function, which makes it possible to know the exact measurement precision for each ability level.

The analyses were performed using the R package *mirt* (31), while the factor analysis can be computed with the function *fa* available in the R package *psych* (32).

## 3 Results

**FIGURE 2:**
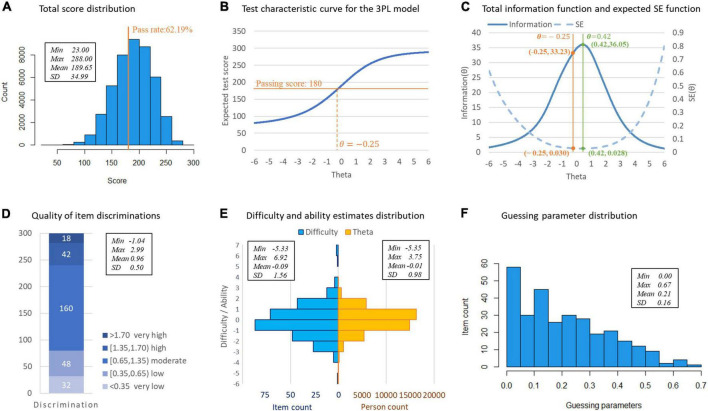
Statistical and item response theory (IRT) analysis results of Standardized Competence Test for Clinical Medicine Undergraduates (SCTCMU). **(A)** Total score distribution. **(B)** Test characteristic curve for the 3PL model. **(C)** Total information function and expected SE function. **(D)** Quality of item discriminations. **(E)** Difficulty and ability estimates distribution. **(F)** Guessing parameter distribution.

The distribution of total observed scores is shown in Figure 2A. Three essential assumptions–unidimensionality, local independence (LD), and the functional form–were checked for using the IRT models. The first and second eigenvalues for EFA were 25.294 and 3.345, respectively, with the ratio between them being 7.562, implying the test was indeed unidimensional. Local independence was assessed using residual correlations and LD statistics. Only seven out of 44,850 item pairs with residual correlation values were greater than |0.20|, and none of the item pairs had an LD statistics value above |5|, suggesting extremely minor inflated reliability estimates (i.e., ignorable problems with construct validity). Figure 2B displays the characteristic curve of the entire test for the 3PL model, which showed the expected score increases monotonically with the level of ability. These results entail that the assumptions of IRT models were satisfied.

The model fit indexes are shown in Table 2. All three models showed an acceptable fit to the data, as all *G*^2^ statistics were insignificant (*p* > 0.05). The LRT showed that the 2PL model fit significantly better than the Rasch model as χ^2^(299) = 232,374,*p* < 0.01, and the 2PL model improves the explanation of the item responses over that of the Rasch model by 3.2% (*R*△_2_=0.032). The 3PL model fit significantly better than the 2PL model as χ^2^(300) =  19,808,*p* < 0.01, and the 3PL model improves the explanation over the 2PL model by 0.28% (*R*△_2_=0.0028). Moreover, AIC and BIC both led to the same conclusion: the 3PL model should be chosen as the calibrating model.

**TABLE 2:** Model fit statistics for the estimated models.

Estimated models	G^2^	*df*	*p*	RMSEA	AIC	BIC	Log likelihood
Rasch	13,586,851	9,999,999,699	1	0	14,536,110	14,538,728	−7,267,754
2PL	13,354,476	9,999,999,400	1	0	14,304,334	14,309,553	−7,151,567
3PL	13,334,668	9,999,999,100	1	0	14,285,126	14,292,955	−7,141,663

Although there were 121 items whose S-χ^2^ values were significant when fitting the 3PL model, this may be due to the excessively a large sample size. The upper bound of RMSEA of all items was only 0.0087, indicating that the items well ensembled the 3PL model. Therefore, the 3PL model was chosen for the coming analysis.

The 3PL model’s item parameter estimates are shown in Figures 2D–F: most items yielded moderate- to-high discrimination power; the difficulty parameters showed an approximately normal distribution and were sufficiently broad to cover a wide ability range of students; the chances of guessing were meager.

As shown in Figure 2B, the pre-determined passing score (180) intersects the test characteristic curve at −0.25 of the model-calibrated scores. The passing score (total score = 180, θ=−0.25) corresponds to test information of 33.23 and an expected standard error (SE) of 0.030 as shown in Figure 2C, which is located close to the peak of the information curve (36.05), equally the lowest measurement errors (0.028), obtained from the model-driven results.

## 4 Discussion

Although existing literature shows many IRT applications to educational assessment in the field of clinical medicine, this study is still unique as it’s a large-scale standardized one, of which the analyses are generally not publicly available. Our results are, therefore, valuable to examiners and/or similar assessment providers; for example, one can compare their item parameters and information volumes with the ranges estimated in the present study to find if his/her target assessment functions better/worse. More importantly, SCTCMU inherits the design and standards from the national licensing exam, and the satisfying analyses do support that this inherence with careful difficulty adjustment is valid; it implies that the essence of the licensing standards can be applied to undergraduates due to medical education’s consistency. Further, the workflow per se can serve as a guide to demonstrate essential steps when investigating the psychometric properties of an examination instead of oversimplifying the analyses.

The evaluation shows the good quality of SCTCMU in terms of model fit and item/test properties. The passing score was set by using Angoff’s method (33) before the actual administration; it turns out to be highly appropriate as it is located close to the lowest measurement errors computed from the model outcomes. A small proportion of items do not meet the LD assumption and present either low discrimination or high guessing parameters, requiring improvements for future item-bank constructions. This work showcases how careful analysis based on IRT can help evaluate the quality of large-scale examination and provides details for replication by applied researchers on how to conduct, interpret, and report IRT analyses.

While the framework of psychometric methods to evaluate the quality of medical education assessments was followed in this paper, further issues concerning in practice should be noticed. First, the range of item parameters (e.g., discrimination and difficulty parameters) used to evaluate item quality is for informational purposes only. It should be interpreted in conjunction with the purpose of the assessment. For example, for criterion-referenced assessments (e.g., admission tests or licensing exams), certain items that examine the content that must be mastered should be retained even if the discrimination parameter is relatively small. Second, purposes of assessment decide the emphasis on the psychometric indicators. The highest measurement accuracy, ideally, should be near the cutoff score for standard-referenced assessments, while other formative assessments (e.g., unit tests) should ensure a high amount of test information on each ability level of the test. Finally, there are different IRT models for data types and test scenarios. For example, the 1PL, 2PL, and 3PL models described in this article could be used for dichotomous scoring items. For polytomous scoring items, the graded response model [GRM, (34)], the partial credit model [PCM; (35)], and the generalized partial credit model [GPCM; (36)] could be used. Further, the multi-faceted Rasch model (37) can deal with situations where the same examinee scored by multiple judges, as in an Objective Structured Clinical Examination (OSCE).

Modern psychometric methods based upon the work of IRT provide a useful approach to the calibration and analysis of medical assessments. IRT can provide standard error of measurement for each ability level, which can facilitate the construct of achievement tests to maximize the measurement precision at the pass-fail point. It can also enable educators to customize formative exams with item difficulties close to an individual’s zone of proximal development (38), making repeated tests more effective. Besides, the metric calibration allows establishing item banks to facilitate continuity and equity in exam standards. However, IRT must be appropriately used, or more harm than good may result. First, all the assumptions need to be met, and the IRT models need to be statistically fit to the observed data. The sample size is another practical issue, as a sizable sample of examinees is required to apply IRT methods successfully.

Due to the collected data set does not contain demographic information, we are unable to test whether the item properties are, in fact, the same across different samples, which involves the issue of test fairness, and can be detected by differential item functioning (DIF) analysis (39). Future SCTCMU will be geared toward computerized adaptive testing platforms with more fine-grained information in its score reporting.

## Data availability statement

The datasets analyzed during the current study are not publicly available but are available from the corresponding author on reasonable request. Requests to access these datasets should be directed to ZJ, jiangzhehan@gmail.com.

## Ethics statement

The studies involving human participants were reviewed and approved by Biomedical Ethics Committee of Peking University (IRB00001052-22070). Written informed consent for participation was not required for this study in accordance with the national legislation and the institutional requirements.

## Author contributions

ZJ and YH developed the study concept and drafted the manuscript. ZJ conducted the literature review and discussion. YH and LX performed the data analysis. JO and TC were involved in drafting and revising the manuscript. All authors contributed to the article and approved the submitted version.
